# Novel Targets and Therapeutic Strategies for Promoting Organ Repair and Regeneration

**DOI:** 10.3390/biom10050749

**Published:** 2020-05-12

**Authors:** Shanmugam Muruganandan, Michael Wigerius

**Affiliations:** 1 Department of Developmental Biology, Harvard School of Dental Medicine, Harvard University, 188 Longwood Avenue, Boston, MA 02115, USA; 2 Department of Pharmacology, Dalhousie University, 1348 Summer Street, North Tower 3rd Floor, Halifax, NS B3H 4R2, Canada; michael.wigerius@dal.ca

## Abstract

Strategies to create functional organs and tissues is of great interest for use in regenerative medicine in order to repair or replace the lost tissues due to injury, disease, as well as aging. Several new treatment options, including stem cell treatments and tissue-engineered substitutes for certain indications, have been approved by Food and Drug Administration (FDA) and are currently available. This special issue will cover new therapies and strategies that are currently being investigated under preclinical and clinical settings.

Organs and tissues in mammalian systems are endowed with a limited regenerative capacity compared to non-mammalian vertebrates and invertebrates [1]. Additionally, tissue-specific variations exist in the regenerative capacity among mammalian organs and tissues [1]. For instance, some tissues, such as the central nervous system (CNS) and heart, show little to no self-renewal capacity, and the liver and pancreas show a slow cell turnover, whereas other tissues, such as intestines, skin, hair follicle, and the skeletal system, display higher levels of renewal, remodeling, and regeneration [1]. The limited regenerative capacity of some tissues and organs poses a serious challenge in devising strategies to restore or repair the lost tissue during injury, aging, and disease. Besides this, even in tissues endowed with high regenerative capacity, it is only up to a certain threshold that these tissues can have the endogenous capacity to regenerate the lost tissue [2]. Thus, approaches to stimulate endogenous stem cells or transplantation of cells and tissues to improve the efficiency of tissue repair is increasingly being appreciated as potential technologies to repair damaged tissues and organs. The discovery of the potential of tissue/cell transplantation to repair and regenerate has accelerated the expectation for clinical application to increase life expectancy, resulting from the replacement of damaged tissues in age-related degenerative diseases. However, clinical application of cell transplantation is limited due to the poor engraftment, proliferation, and differentiation potential of transplanted cells under clinical conditions [3]. In recent years, some of these limitations are being attempted to be overcome by combining complementary tissue engineering technologies, such as the application of genetically modified cells, biochemical factors, biomaterials, and gene therapy, to regenerate biological tissues.

This special issue of *Biomolecules* is dedicated to highlighting the current contributions of research on therapeutic targets and bioengineering strategies to promote organ repair and regeneration. Strategies for developing functional organs and tissues from cell culture models, tissue-engineered substitutes, biomolecules from cells, molecular and cell biological approaches, surgical applications, and stem cell therapies are the major contributions to this special issue. A large number of studies have developed translational approaches to replace damaged cells, tissues, and organs [4,5,6,7,8,9,10,11,12,13,14,15,16]. However, devising novel strategies for organ-specific regeneration faces major limitations due to critical challenges in accurately replicating complex organ-specific features, such as the arrangement of cells and matrix into three-dimensional (3-D) structures of the organ. Recent studies have also attempted to overcome this limitation by replicating 3-D organ structures in tissue cultures and tissue-engineered substitutes for the study of organ repair and remodeling [4,10,17,18]. Abdulghani and Mitchel ‘Biomaterials for In Situ Tissue Regeneration: A Review’ provide an overview of novel strategies using structured scaffolds to induce the regeneration of some native tissues, such as blood vessels, bone, and cartilage [10]. The recent findings on different strategies and tissue-specific biomaterial scaffolds to completely fill the complex structures of the 3-D anatomical defect, create the microenvironment necessary for recruitment of precursor stem cells in the host tissue, incorporate the signals essential for cell proliferation, differentiation, and tissue regeneration, in addition to providing the structural support until the end of the repair process are summarized in the context of a spectrum of organs and tissues with varying degrees of regenerative capacity [10]. The contributions by Sultankulov et al. ‘Progress in the Development of Chitosan-Based Biomaterials for Tissue Engineering and Regenerative Medicine’, by Kazimierczak et al. ‘Development and Optimization of the Novel Fabrication Method of Highly Macroporous Chitosan/Agarose/Nanohydroxyapatite Bone Scaffold for Potential Regenerative Medicine Applications’, and by Saberi et al. ‘Electrically Conductive Materials: Opportunities and Challenges in Tissue Engineering’ identify strategies and update the progress on using new materials for tissue engineering [4,5,6]. Saberi et al. review recent findings on electrically conductive materials for tissue engineering. They discuss novel strategies using electrically conductive materials to solve problems associated with the conventional scaffolds that cannot probe physicochemical and biological microenvironments. Scaffolds with electrical, mechanical, and chemical properties that meet the conductivity of tissues ranging from ventricular muscle, nerve, lung, cardiac, and skeletal muscle for the promotion of tissue-specific regeneration are elaborately discussed [5]. Sultankulov et al. review the progress made in the development of chitosan-based scaffolds in wound healing and bone and cartilage regeneration, besides its use for drug delivery applications [6]. Kazimierczak et al. developed a novel biomaterial composed of polysaccharide matrix (chitosan–agarose) reinforced with nanohydroxyapatite that mimics the 3-D bone structure with macroporosity to facilitate osteoblast growth and bone regeneration [4].

The contributions by Urkasemsin et al. ‘Strategies for Developing Functional Secretory Epithelia from Porcine Salivary Gland Explant Outgrowth Culture Models’ propose a method to develop transplantable human salivary gland secretory epithelia for patients with salivary gland hypofunction and xerostomia [8]. Similar cell-based strategies to assess the therapeutic potential of stem cells for the creation of functional organs and tissues have been developed by Sabbah et al. ‘Predicting Angiogenesis by Endothelial Progenitor Cells Relying on In-Vitro Function Assays and VEGFR-2 Expression Levels’ and by Mukhamedshina et al. ‘Mesenchymal Stem Cell Therapy for Spinal Cord Contusion: A Comparative Study on Small and Large Animal Models’.

Sabbah et al. describe the need for a potency assay in order to assess the transplantability of endothelial progenitor cells for therapeutic applications. Endothelial cell damage is an underlying factor in a number of vascular disorders, with the concept of endothelial progenitor cell (EPC) therapy offering attractive treatment to these disorders [19,20]. The approach involves transplantation of endothelial progenitor cells (EPCs) isolated from the patient’s own tissue to replenish damaged endothelial cells and repair injured tissue. While much progress has been made in our understanding of the molecular mechanisms that underlie revascularization, clinical application of EPC therapy is still facing a number of challenges [21]. This includes the identification of EPC populations with a strong regenerative capability that may offer suitable options for clinical use. For example, in an attempt to isolate EPCs from 18 blood samples for potential applications in regenerative medicine, Huizer et al. (2017) found that the EPCs from two samples alone were endowed with abilities to generate late-outgrowth EPCs that are superior in their therapeutic angiogenic potential compared to EPCs from other samples [22]. In this issue, Sabbah et al. (2019) explore the ability of subsets of EPCs collected from the blood samples of healthy volunteers to form new vessels before and after transplantation [9]. By measuring known parameters for EPC growth in vitro and comparing blood vessel density following transplantation in a mouse model, the authors identified significant variations in vessel density and, thus formation, among the different donors. While a number of molecular factors predicting vessel formation have been identified, Sabbah et al. focused on the mRNA levels of the vascular endothelial growth factor receptor 2 (VEGFR-2). They found that high VEGFR-2 expression correlated with EPC subsets that were particularly efficient in promoting vessel formation consistent with earlier observations that have implicated VEGFR-2 in different aspects of angiogenesis. Thus, this study highlights the functional variability that exists among EPCs isolated from different sources and the importance to predict their angiogenic capacity. The authors further highlight methods to predict vessel formation and thereby vascular regeneration by EPCs following transplantation that may indeed be an attractive option in personalized medicine [9].

Using small and large animal spinal cord injury (SCI) models, Mukhamedshina et al. [11], in their study entitled ‘Mesenchymal Stem Cell Therapy for Spinal Cord Contusion: A Comparative Study on Small and Large Animal Models’, provide evidence for neuronal regeneration by the application of adipose tissue-derived mesenchymal stem cells (AD-MSCs) embedded in fibrin matrix at the site of SCI in rats and pigs. Their observations in a porcine model of SCI support the potential therapeutic application for AD-MSC transplantation in treating subacute paraplegia in large animals and humans. However, it is generally accepted that aging adversely affects MSC functions, such as their replicative potential and properties, including immunomodulatory and secretory profiles, by stimulating senescence in vivo [15]. MSCs from advancing donor age are reported to be senescent with limited abilities to proliferate and differentiate that compromise their regenerative potential. The paper by Neri et al. reviews recent findings on MSC aging mechanisms and strategies to overcome senescence in MSCs. In particular, the identification of senolytic and senomorphic agents to clear and/or revert senescent cells is of great interest for their regenerative potential by targeting senescent MSCs and as well as for their general use in treating age-related pathologies [15].

The study by Triaca et al. investigates the therapeutic potential of small stable nerve growth factor (NGF) peptides as tropomyosin receptor kinase A (TrkA) agonists for treating pathologies of the central and peripheral nervous system [23]. NGF is the founding member of a family of extracellularly secreted growth factors in the nervous system that collectively are called neurotrophins, which also include brain-derived neurotrophic factor, neurotrophin-3, and neurotrophin 4/5 [24]. The TrkA expressed on the surface of neurons in the central and peripheral nervous systems is the primary target for NGF and failure of downstream NGF/TrkA signaling has been linked to various neurodegenerative disorders [24]. Understanding how nerve growth factor (NGF) promotes the differentiation and survival of neurons has therefore been a central aim in neurobiology research for many years. Additionally, it has become clear that NGF exerts critical roles outside of the nervous system, providing a significant impact on the immune and endocrine systems as well. With an ability to reduce cellular damage in different biological systems, NGF may offer potential therapeutic options in regenerative medicine [24].

The production of NGF by recombinant methods has made it possible to test its clinical efficacy, but basic principal limitations have remained unsolved. A critical limitation is the inability of NGF to cross the blood–brain barrier (BBB) upon systemic administration of the protein [25,26]. Consequently, it has proven difficult to design molecules that both pass through the BBB and efficiently potentiate TrkA activity. An alternative and possibly promising approach has been to design small peptide ligands based on the N-terminal region in NGF, which binds the extracellular portion of the TrkA receptor. In this issue, Triaca et al. investigated the ability of peptide derivatives of human NGF (hNGF) to activate TrkA receptor signaling [23]. The authors found that a small peptide, encoding amino acids 1-14 in the N terminal region NGF (hNGF1-14), was able to trigger established TrkA pathways with high efficacy. In cholinergic and dorsal root ganglia neurons, hNGF1-14 stimulated the expression of markers driving neuronal activity and survival, indicating that the peptide can retain the biological activity of native NGF [23]. Incubation with an acetylated counterpart of hNGF1-14 (Ac-hNGF1-14) showed even greater potency in stimulating TrkA signaling, suggesting that enhancing the stability of the peptide may affect the formation of the ligand–receptor complex. Additional electrophysiological experiments demonstrated that Ac-hNGF1-14 enhanced the frequency of cholinergic cultures, indicating that the derivative is capable of enhancing the spontaneous firing of excitatory neurons. Nonetheless, there are still many questions that remain to be answered regarding the safety, dose, and timing of hNGF1-14 in various biological disorders. Whatever the answers, the data in hand suggest that hNGF1-14 and its derivatives may offer an efficient delivery strategy in vivo. The results also strengthen the view that peptidomimetics may be an important tool in the development of compounds with therapeutic agonist activity.

A major complication following the implantation of a biomaterial or prosthesis is the incidence of infections caused by growth of bacterial biofilms on prosthetic surfaces. In addition, despite the use of antibiotics to modify prostheses, implant-associated infections still remain challenging due to the development of bacterial resistance. To address this issue, Szałapata et al. examined the prospect of serine protease inhibitors (PIs), immobilized on prosthetic surfaces widely used in regenerative medicine, to prevent bacterial growth [27]. Initially, they monitored the activity of the PIs at different concentrations, pH values, and temperatures along with the concentration of cross-linker to establish the conditions for surface immobilization. The authors then exploited the ability of the PIs to prevent the growth of bacterial biofilm that are commonly reported to adhere to prosthetic implants. They reported that the covalent immobilization of PIs on the surface of biomaterials introduced significant changes in their surface structure with anti-adhesive and bacteriostatic/bactericidal properties [27]. Taken together, these results suggest that the application of the investigated enzymes under the specified conditions for immobilization has the potential to reduce bacterial invasiveness and may offer a novel treatment for patients with prostheses.

In summary, these articles on the emerging therapies in regenerative medicine encompass the use of stem cell-based strategies, approaches to produce functional organs and tissues, tissue-engineered substitutes, biomolecules, recombinant peptides, and other molecular and cell-based approaches to promote organ and tissue repair. Great advances have been made in the development of new methods and technologies that implicate an exciting future for cell and tissue therapies in regenerative medicine and biomaterial-based tissue engineering. These new advances will pave the way for a better understanding of mechanisms of organ and tissue loss during injury and diseases and for the design of strategies to rebuild damaged tissues for therapeutic purposes.
